# Gastric Leiomyosarcoma as a rare cause of gastric outlet obstruction and perforation: a case report

**DOI:** 10.1186/1756-0500-7-479

**Published:** 2014-07-29

**Authors:** Elroy P Weledji, George Enoworock, Marcelin Ngowe Ngowe

**Affiliations:** 1 Department of Surgery, Faculty of Health Sciences, University of Buea, PO Box 126 Limbe, S.W. Region, Buea, Cameroon; 2 Department of Pathology, Faculty of Health Sciences, University of Buea, Buea, Cameroon

**Keywords:** Gastric outlet obstruction, Leiomyosarcoma, Gastrointestinal stromal tumours

## Abstract

**Background:**

Gastrointestinal stromal tumours are the most common mesenchymal malignancies of the gastrointestinal (GI) tract and gastric leiomyosarcoma represent 1-3% of gastric malignancies.

**Case presentation:**

We report a case of a 69-year- old black African man who presented with a rare cause of gastric outlet obstruction and duodenal perforation. A Billroth- II gastrectomy was performed and histology confirmed a gastric leiomyosarcoma.

**Conclusions:**

It is important to identify the gastric leiomyosarcoma which is a variant of the more common malignant gastrointestinal stromal tumours as the pathogenesis and management are currently well established. As the facilities for differentiating these are not easily available in resource-limited areas gastrointestinal stromal tumours may remain underdiagnosed and undertreated.

## Background

Gastrointestinal stromal tumours (GISTs) are the most common mesenchymal (non-epithelial) malignancies of the gastrointestinal (GI) tract [1,2]. Most arise in the stomach (leiomyosarcoma) representing 0.1-3% of gastric malignancies, or small intestine, and less frequently in the oesophagus, mesentery, omentum, colon or rectum [1]. Historically, GISTs were considered to be of smooth muscle origin and were generally regarded as benign (leiomyomas) or malignant (leiomyosarcomas). However, only a minority of stromal tumours have the typical features of smooth muscle with some having a more neural appearance and others appearing undifferentiated [3]. The discovery of CD34 expression (80-90%) and the receptor tyrosine kinase KIT (CD117) in many GISTs suggested that they were a specific entity distinct from smooth muscle tumours [4]. This has led to the widely accepted classification of mesenchymal tumours of the GI tract into GISTS, true smooth muscle tumours, and, far less frequently, true schwann cell tumours [3]. Appropriate management of gastrointestinal mesenchymal tumours requires accurate diagnosis and should involve a multidisciplinary approach. Overall, the prognosis of leiomyosarcoma is poor, death often resulting from local spread and/or metastases.

## Case presentation

**Figure 1:**
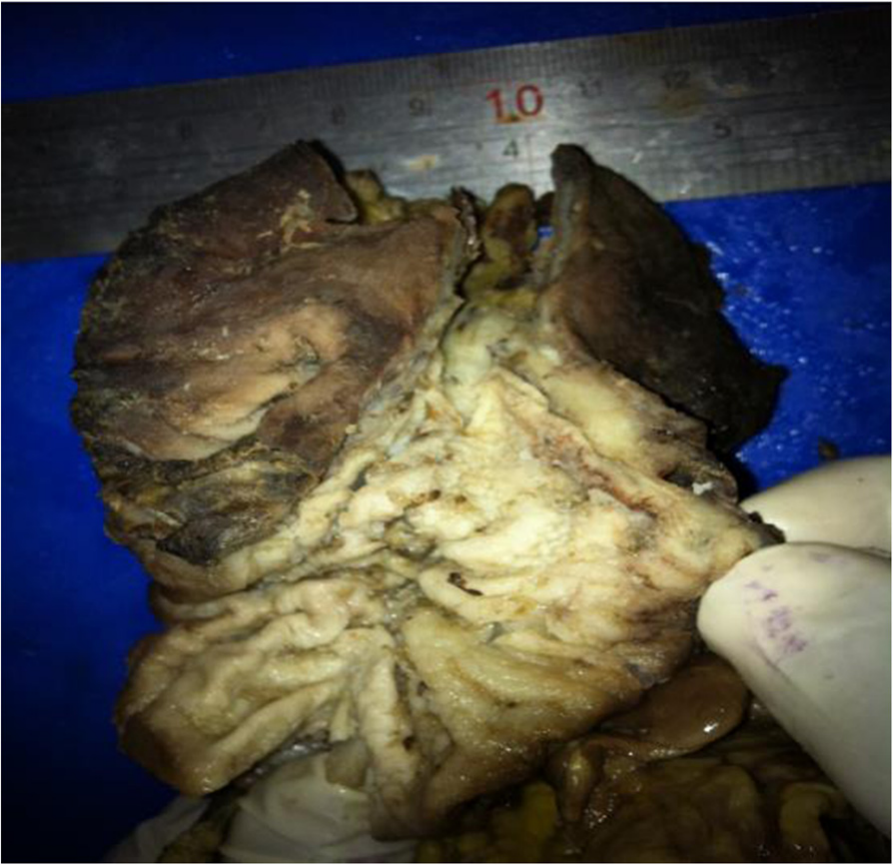
Large gastric leiomyosarcoma (>8 cm) - high chance of dissemination.

**Figure 2:**
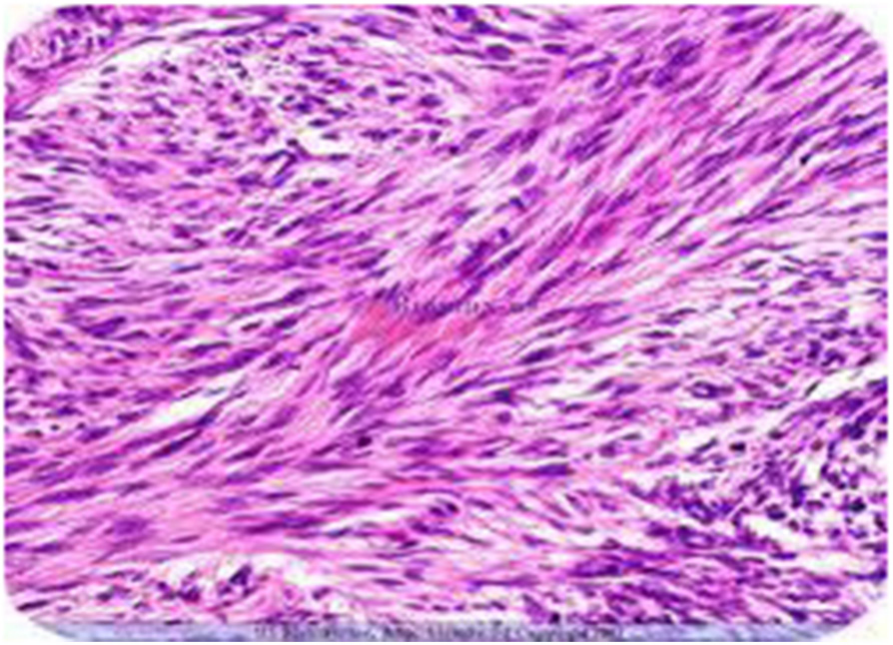
**Photomicrograph showing the spindle cells of leiomyosarcoma with multiple abnormal mitosis.** (haematoxylin and eoisin stain).

A 69-yr- old African man was admitted as an emergency with a 2- week history of symptoms of gastric outlet obstruction. He had postpandrial vomiting associated with upper abdominal bloatedness and complained of a constant epigastric pain in the past 48 hrs. He had no haemetemesis nor melaena. There was no antecedent history of peptic ulcer disease nor a past history of similar symptoms. He had anorexia, weight loss and constipation. On physical examination he appeared pale and dehydrated. BP was 100/80 mmHg, HR 96/min, temperature 36.5°C. The distension of the upper abdomen was consistent with a dilated stomach. He had no definite signs of peritonism (rebound tenderness, guarding, abdominal rigidity) but mild tenderness on deep palpation of an epigastric mass. The differential diagnosis included a malignant gastric outlet obstruction or an acquired pyloric stenosis from chronic peptic ulcer disease. The haemoglobin level was 10 gm/l, a white cell count was not available but the urea and electrolytes were within normal limits. An erect chest x-ray showed no air under the diaphragm. A plain abdominal x-ray revealed a grossly dilated stomach with food contents. An ultrasound scan (U/S) suggested a pyloric tumour. Following resuscitation for 48 hrs with i/v fluids, antibiotics and nasogastric suction, he consented to a laparotomy. At laparotomy there was pus emanating from the left subhepatic space. The stomach was grossly dilated with a 3-4 cm anterior perforation of the 1st part of the duodenum with surrounding necrosis overlying the common bile duct and gastroduodenal artery. There was seeding of tumour deposits into the serosa and adjacent omentum. The liver appeared normal on palpation. Kocherization of the duodenum allowed a difficult Billroth II partial gastrectomy and a retrocolic gastrojejunostomy fashioned. An open right subhepatic drain was inserted. Intraoperatively he was haemodynamically unstable with hypotension requiring a transfusion of 2 units of blood. Postoperatively, he remained haemodynamically unstable (BP 93/73 mmHg, pulse 97/min,) with associated sweating and oliguria. He was apyrexial but hypoglycaemic with a fasting blood sugar (FBS) of 10.6 g/dl (n 14-18 g/dl). He responded to fluid resuscitation and glucose repletion. He tolerated liquid diet on the 5th post operative day and his haemoglobin level was 12.6 g/l. On the 7th postoperative day he suddenly became clammy and died. A postmortem was not done. The histology reported a partial gastrectomy specimen measuring 10.0 x 8.0 x 7.0 cm, rough surfaced, in folds, brownish-yellow colouration and elastic in consistency. Microscopy showed a proliferation of fusiform cells with large, central hyperchromatic nuclei, mottled chromatin and abundant mitosis showing smooth muscle differentiation invading a myxoid and fibro-fatty stroma. This was consistent with a malignant gastrointestinal stroma tumour (malignant GIST) of the leiomyosarcoma type (Figures 1 and 2).

## Discussion

Although almost all perforated gastric ulcers can be effectively managed by laparotomy and omental patch repair, intraoperative biopsy and follow-up endoscopy with repeat biopsy is essential to avoid an underlying malignancy. Large perforation of gastric ulcers usually require distal gastrectomies as in this case [5]. The histology of the resected specimen revealed a malignant GIST (leiomyosarcoma). Gastric leiomyosarcomas represent 10–15% of mesenchymal tumours and the age distribution and clinical presentation are similar to malignant gastrointestinal stromal tumours (GISTs). The associated systemic symptoms such as fever, night sweats and weight loss are very rare in other sarcomas [1]. The median age at diagnosis is 50-60 years with a slight male predominance. They are often clinically silent until they reach a large size, bleed or rupture [6,7]. Thus, the diagnosis is rarely made preoperatively. Since in many cases the mucosa is normal, a definitive diagnosis for resectable tumour is often made after surgery [7-9]. Seeding of tumour deposits into the serosa or omentum is almost invariably a sign of malignancy. In this case, the large tumour size greater than 8 cm and mucosal perforation indicated rapid tumour growth and more likely to be associated with disseminated disease [1,3]. The optimisation of this patient’s fitness for surgery was compromised by the emergency presentation with gastric outlet obstruction and localized peritonitis. This was exacerbated by the lack of adequate and appropriate postoperative care usually rendered in a high dependency or intensive care unit especially in this patient requiring cardiac and renal support. These are the short-comings of performing major surgery in poor resourced areas. It is known that cancer deaths in hospital involves patient factors, tumour factors and surgeon-related factors [10]. Both tumour (large size with obstruction and perforation) and patient factors were not favourable. The probable immediate cause of death was cardiac failure as he remained cardiovascularly unstable perioperatively.

In resourced areas, initial diagnosis of malignant GIST is based on imaging. Endoscopic ultrasonography (EUS) especially of the oesophagus, stomach, duodenum and anorectum can confirm the diagnosis of small lesions less than 2 cm. For large tumours, computed tomography (CT) of chest, abdomen and pelvis would assess primary tumour extension and stage for metastases. Percutaneous (US or CT -guided) or laparoscopically- guided biopsies are not used in resectable disease due to the risk of tumour rupture or seeding unless it may result in a change of treatment [8]. Laparoscopy may be considered to stage for peritoneal and distant spread of disease [7,9]. For patients with unresectable and/or metastatic tumours, an endoscopic or percutaneous biopsy is taken for a definitive diagnosis before treatment.

As the morphological spectrum of GISTs is wider than previously recognized, macroscopic examination of the site of the resected tumour, with an adequate sampling for histological examination and for immunohistochemistry should be performed [9]. In this case, the histopathological diagnosis of a malignant GIST would have been supported by a positive expression of CD34 or CD117 [4,11]. Evaluation of respectability of gastrointestinal stromal tumour is determined by the surgeon and depends on the stage and the individual patient’s fitness for surgery [7,8]. As they rarely metastasise to lymph nodes, extended lymphadenectomy is seldom warranted. En bloc resection of involved adjacent organs is necessary for oncological clearance [8,9]. As metastasis is primarily haematogenous, the five year survival following surgical (RO) resection is 37-54% [5]. Neither palliative or adjuvant radiotherapy nor standard chemotherapy has been shown to be of benefit [7,8]. The early results suggest that molecular therapy with the tyrosine kinase inhibitor, imitanib mesylate, may play an important role as adjuvant therapy following GIST resection. It increases recurrence –free survival as shown by contrast enhanced CT scanning [12,13].

## Conclusion

It is important to identify the gastric leiomyosarcoma which is a variant of the more common malignant gastrointestinal stromal tumour (GISTs) whose pathogenesis and management are currently well established. As the facilities for differentiating these are not easily available gastrointestinal stromal tumours may remain underdiagnosed and undertreated in resource-limited areas.

### Consent

“Written informed consent was obtained from the next of kin to the deceased for the case report and accompanying images to be published. A copy of the written consent is available for review by the editor-in- chief of this journal”.

## Competing interests

The authors declare that they have no competing interests.

## Authors’ contributions

EPW was the surgeon and carried out the design of the case report and drafted the manuscript; GE was the pathologist who provided the histology report of the resected specimen and participated in the article’s design; MNN participated in the design and coordination. All authors read and approved the final manuscript.
